# Association of metabolic indices with in-stent restenosis and its angiographic severity after drug-eluting stent implantation: a case-control study

**DOI:** 10.3389/fendo.2026.1952213

**Published:** 2026-09-21

**Authors:** Fabrice Yves Ndjana Lessomo, Yinhua Luo, Di Fan, Zhanpeng Li, Yingying Hu, Zhiquan Wang, Zhibing Lu

**Affiliations:** 1 Department of Cardiology, Zhongnan Hospital, Wuhan University, Wuhan, Hubei, China; 2 Hubei Provincial Clinical Research Center for Cardiovascular Intervention, Wuhan, Hubei, China; 3 Institute of Myocardial Injury, Wuhan, Hubei, China; 4 Cardiovascular Center, Taikang Tongji (Wuhan) Hospital, Taikang Clinical College of Wuhan University, Wuhan, Hubei, China

**Keywords:** atherogenic index of plasma, coronary intervention, in-stent restenosis, metabolic markers, METS-IR, sex differences, TyG index

## Abstract

**Background:**

Metabolic abnormalities may contribute to in-stent restenosis (ISR) after percutaneous coronary intervention, but the comparative associations of commonly used metabolic indices with ISR within the same patient population remain incompletely characterized.

**Objective:**

To compare the associations of the triglyceride-glucose (TyG) index, atherogenic index of plasma (AIP), and metabolic score for insulin resistance (METS-IR) with angiographically detected ISR and its severity, and to explore potential heterogeneity according to sex and clinical presentation.

**Design:**

Retrospective, propensity-score-matched case-control study of patients undergoing follow-up coronary angiography between 2019 and 2024.

**Participants:**

The propensity-score-matched cohort comprised 774 patients, including 387 patients with ISR and 387 matched controls without ISR. Angiographic severity analyses were performed among the 387 matched patients with ISR.

**Main measures:**

TyG, AIP, and METS-IR were assessed separately in multivariable models. Model discrimination was evaluated using the area under the receiver operating characteristic curve (AUC), paired DeLong testing, net reclassification improvement (NRI), and integrated discrimination improvement (IDI). Sex- and clinical-presentation analyses were considered exploratory.

**Key results:**

The baseline clinical and procedural model yielded an AUC of 0.716 (95% CI 0.672–0.763) for ISR occurrence. Addition of TyG increased the AUC to 0.756 (95% CI 0.708–0.803; ΔAUC +0.040; paired DeLong p=0.003), while addition of AIP increased the AUC to 0.772 (95% CI 0.726–0.806; ΔAUC +0.056; p<0.001). Addition of METS-IR did not improve discrimination (AUC 0.716, 95% CI 0.671–0.748; p=0.642). Bootstrap analyses supported the incremental discrimination associated with TyG and AIP but not METS-IR. For ISR severity, addition of TyG or AIP produced only modest increases in AUC, neither of which was significant by paired DeLong testing. Formal interaction analyses did not demonstrate significant effect modification by sex, and analyses according to clinical presentation were limited by small subgroup sizes.

**Conclusions:**

In this retrospective matched cohort, TyG and AIP provided incremental discriminatory information for angiographically detected ISR beyond conventional clinical and procedural factors, whereas METS-IR provided little additional value. Evidence regarding angiographic severity was less robust, and findings across sex and clinical-presentation subgroups should be considered exploratory. Prospective external validation is required before these indices can be considered for clinical risk stratification.

## 1 Introduction

Percutaneous coronary intervention (PCI) with drug-eluting stents (DES) remains the cornerstone of treatment for obstructive coronary artery disease (CAD) (1). Despite continuous advancements in stent platform technologies and polymer biocompatibility that have drastically reduced early neointimal hyperplasia, in-stent restenosis (ISR) persists as a challenging complication that compromises long-term clinical outcomes and frequently necessitates target lesion revascularization (2). The pathogenesis of modern DES restenosis is multifactorial, driven largely by chronic localized inflammation, delayed endothelial healing, smooth muscle cell migration, and the progressive development of neo-atherosclerosis (3, 4).

While traditional cardiovascular risk factors such as diabetes mellitus, dyslipidemia, and smoking are acknowledged contributors to stent failure, they do not fully capture the observed heterogeneity in ISR incidence (5). Growing interest has therefore focused on readily available metabolic indices that integrate abnormalities in glucose and lipid metabolism. The triglyceride-glucose (TyG) index, atherogenic index of plasma (AIP), and metabolic score for insulin resistance (METS-IR) have been proposed as inexpensive surrogate markers of insulin resistance and atherogenic metabolic dysfunction (6–8). These indices reflect partially overlapping but biologically distinct aspects of cardiometabolic risk. TyG principally integrates fasting triglyceride and glucose concentrations, AIP reflects the relationship between triglycerides and high-density lipoprotein cholesterol, whereas METS-IR combines glucose, triglycerides, high-density lipoprotein cholesterol, and body mass index. Elevated values of these metabolic indices have been associated with adverse vascular phenotypes, including arterial stiffness, endothelial dysfunction, and cardiovascular events (9). Nevertheless, their comparative associations with ISR within the same DES-treated population remain insufficiently characterized, and it is uncertain whether one index provides more informative discrimination than the others when assessed against the same clinical and procedural background. Indices such as the Triglyceride-Glucose (TyG) index, the Atherogenic Index of Plasma (AIP), Elevated values of these indices correlate strongly with increased arterial stiffness, endothelial dysfunction, and future cardiovascular events in primary prevention cohorts (9).

Potential heterogeneity by sex represents an additional source of uncertainty. Sex differences have been described in the clinical presentation, vascular biology, risk-factor profile, and outcomes of CAD after PCI (10, 11). Women may also differ from men in inflammatory responses and lipid metabolism, partly because of hormonal and metabolic influences (12). However, whether the associations of TyG, AIP, and METS-IR with ISR differ according to sex has not been adequately established using formal interaction analyses. Clinical presentation may also influence the probability of ISR detection because follow-up angiography may be performed either for symptoms or as routine surveillance; consequently, presentation-related analyses require cautious interpretation given the potential for angiographic ascertainment bias.

Against this background, the present study aimed to directly compare TyG, AIP, and METS-IR within the same study population and evaluate their associations with angiographically detected ISR after DES implantation using a propensity-score-matched case-control design. We further examined their associations with angiographic ISR severity among patients with documented ISR and with the timing of angiographic ISR detection, recognizing that the date of angiographic diagnosis does not necessarily represent the biological onset of restenosis. Finally, we explored whether the associations between these metabolic indices and ISR varied according to sex and clinical presentation, with subgroup findings considered exploratory. By comparing three commonly accessible metabolic indices within a common analytical framework, this study sought to clarify their relative associations with ISR while distinguishing association and discrimination from prospective prediction or causal inference.

## 2 Materials and methods

### 2.1 Study design and population

This retrospective case-control study identified adult patients aged 18 years and older at Zhongnan Hospital, Wuhan University, with prior drug-eluting stent (DES) implantation who underwent follow-up coronary angiography between May 2019 and December 2024. Follow-up coronary angiography was performed because of clinical indications or routine surveillance. Patients with angiographically documented in-stent restenosis (ISR) were included as cases. Controls were patients without ISR, without typical coronary chest pain, and without significant coronary stenosis on follow-up angiography. Because the control group was defined as having no ISR, no typical coronary chest pain, and no significant coronary stenosis, routine follow-up was expected to be considerably more frequent among controls than among ISR cases. The case-selection process is detailed in the ANNEX.

Patients were excluded if they had a history of coronary artery bypass grafting (CABG), acute ST-segment elevation myocardial infarction or cardiogenic shock during follow-up, incomplete data required for calculation of the metabolic indices, advanced hepatic or renal dysfunction, active malignancy, or other severe comorbidities that could substantially affect the study assessment. The unit of analysis was the patient.

The presentation during follow-up appointments was recorded in two ways: as routine follow-up (FU) when the patient did not exhibit any significant or major symptoms at the time of admission, and as symptomatic when the patient came in with suggestive symptoms. This also applied when the visit was not related to the standard institutional surveillance (12 months after PCI) but was prompted by any discomfort experienced by the patient.

The second-generation drug-eluting stents, including sirolimus-, everolimus-, and zotarolimus-eluting platforms, were the most commonly used types in patients. The Institutional Review Board waived informed consent requirements due to the retrospective design (IRB No. 2025005).

### 2.2 Angiographic assessment and definitions

Follow-up coronary angiography was performed due to clinical indications (symptom-driven) or routine surveillance. Clinical Presentation: Categorized broadly into Symptomatic vs. Routine Follow-Up, and detailed further by specific symptoms (e.g., chest pain, palpitations, fatigue).

ISR Occurrence was defined as ≥50% diameter stenosis within the stented segment or within 5 mm proximal or distal to the stent edges. In patients with more than one implanted stent or more than one re-stenotic segment, the lesion with the greatest percentage diameter stenosis on follow-up angiography was considered the index lesion for the primary angiographic analysis.

ISR Severity: ISR severity was stratified into Less Severe (50% to 75% diameter stenosis) and Very Severe (>75% diameter stenosis). This classification was chosen based on clinical relevance, as >75% stenosis is more likely to be flow-limiting and associated with symptoms requiring repeat revascularization (2).

Timing of Detection: Stratified as Early Detection (≤12 months post-index PCI) and Late Detection (>12 months post-index PCI). Because the exact biological onset of restenosis cannot be determined from retrospective angiographic follow-up, this variable is referred to throughout the manuscript as the timing of angiographic ISR detection, rather than the timing of ISR development.

Quantitative coronary angiography (QCA) was used for all assessments, measuring reference vessel diameter, minimum luminal diameter, percent diameter stenosis, and lesion length. The process involved a validated digital analysis system in the cardiac catheterization laboratory, with experienced interventional cardiologists independently reviewing the measurements without knowledge of the metabolic-index values. Standard QCA techniques were followed, and any disagreements were resolved by consensus. Due to the retrospective nature of the study, a formal interobserver reproducibility assessment was not performed.

### 2.3 Data collection and metabolic indices

Data collected included demographic and clinical variables (age, sex, body mass index, history of hypertension, diabetes mellitus, and smoking status), procedural and lesion characteristics (drug-eluting stent type, stent diameter and length, number of stents, and presence of stent overlap), and key biomarkers (fasting triglycerides, HDL cholesterol, glucose, high-sensitivity troponin I) measured during the hospitalization at follow-up angiography.

Fasting plasma glucose, triglycerides, HDL-C, and other biochemical parameters measured at this admission were used to calculate TyG, AIP, and METS-IR.

Triglyceride-Glucose Index (TyG):


$$\text{TyG}=\text{ln}(\frac{\text{TG }(\text{mg}/\text{dL})\times \text{FPG }(\text{mg}/\text{dL})}{2})$$

Atherogenic Index of Plasma (AIP):


$$\text{AIP}=\text{lo}{\text{g}}_{10}(\frac{\text{TG }(\text{mg}/\text{dL})}{\text{HDL}-\text{C }(\text{mg}/\text{dL})})$$

Metabolic Score for Insulin Resistance (METS-IR):


METS-IR=[ln(2×FPG)+TG]×BMI /ln(HDL−C)

Because TyG, AIP, and METS-IR are strongly intercorrelated, they were evaluated separately in subsequent multivariable analyses.

The AIP formula was calculated as log_10_(TG/HDL-C) where TG and HDL-C are expressed in mg/dL. This approach is consistent with the original validation by Dobiásová and Frohlich (7), who demonstrated that the log transformation of the TG/HDL-C ratio (in mg/dL) correlates strongly with LDL particle size.

METS-IR was calculated using the original formula described by Bello-Chavolla et al. (8): [ln(2 × FPG) + TG] × BMI/ln(HDL-C), where FPG is in mg/dL, TG is in mg/dL, HDL-C is in mg/dL, and BMI is in kg/m^2^.

### 2.4 Propensity score matching

To reduce baseline differences between patients with and without ISR, propensity score matching (PSM) was performed using a multivariable logistic regression model that incorporated prespecified covariates: age, sex, statin use, Gensini score, stent length, vessel size, hypertension, diabetes, and smoking status. A nearest-neighbor algorithm without replacement was applied at a 1:1 ratio, using a caliper of 0.062, corresponding to 0.20 standard deviations of the propensity score logit. This approach yielded 387 matched pairs (N = 774). Covariate balance was evaluated using standardized mean differences (SMDs), with an absolute SMD <0.10 considered indicative of adequate balance. A sensitivity analysis using a tighter caliper of 0.050 produced 381 matched pairs and preserved the primary findings. Clinical presentation was not included in the PSM model because it was considered to be on the causal pathway for angiography indication; however, it was explored in sensitivity analyses restricted to patients with comparable indications for follow-up angiography.

### 2.5 Statistical analysis

Continuous variables are presented as mean ± SD or median (IQR), while categorical variables are shown as frequencies (%). Normality was assessed using the Shapiro-Wilk test. For group comparisons, Student’s t-test, one-way ANOVA, Mann–Whitney U test, or χ^2^ test was employed as appropriate.

The primary analysis of ISR occurrence was performed in the propensity-matched cohort of 774 patients (387 matched pairs). Because propensity matching created a paired structure, binary logistic regression and conditional logistic regression with matched-pair subclass as the stratum was also used to evaluate the association of each metabolic index with ISR occurrence. TyG, AIP, and METS-IR were entered into separate models because of strong multicollinearity among the three indices. Models were adjusted for age, sex, hypertension, diabetes, smoking status, statin use, stent length, and vessel size.

ISR severity was first analyzed among 387 patients with documented ISR because severity is defined only after ISR has occurred. Multivariable binary logistic regression was used to compare Very Severe versus Less Severe ISR, with each metabolic index evaluated separately and adjusted for the same clinical and procedural covariates; see ANNEX.

The analysis of ISR timing was also restricted to the unmatched 820 patients with ISR. Multivariable logistic regression was used to evaluate Early (≤12 months) versus Late (>12 months) angiographic ISR detection. Spearman’s rank correlation was used to assess the relationship between each metabolic index and the continuous time from index PCI to angiographic ISR detection. A sensitivity model additionally adjusted for Gensini score. These analyses reflect the timing of angiographic detection and not the biological onset of ISR.

Model diagnostics included assessment of multicollinearity using variance inflation factors (VIFs), with VIFs <4 considered acceptable (ANNEX).

Model discrimination was assessed by measuring the area under the receiver operating characteristic curve (AUC). AUC values are reported with 95% confidence intervals. Because the ROC curves were derived from the same patients, comparisons between the baseline and extended models were performed using paired DeLong tests. Differences in AUC were additionally evaluated using 1,000 matched-pair bootstrap resamples to obtain 95% confidence intervals.

Net Reclassification Improvement (NRI) and Integrated Discrimination Improvement (IDI) were calculated in comparison with the baseline clinical and procedural model. Confidence intervals and p-values for NRI and IDI were obtained using 1,000 matched-pair bootstrap resamples. Internal validation was also performed using 1,000 matched-pair bootstrap samples to estimate optimism-corrected model discrimination.

Subgroup analyses were performed by sex and clinical presentation. Formal sex × metabolic-index interaction terms were used to assess possible sex heterogeneity rather than relying only on separate male and female models. Clinical-presentation analyses were considered exploratory because of the substantial imbalance in angiographic indication. A same-indication sensitivity analysis was therefore performed among patients undergoing routine follow-up. Because some presentation strata contained sparse outcome counts, Firth penalized logistic regression was used where appropriate.

For families of related subgroup and interaction analyses, the Benjamini-Hochberg false-discovery-rate procedure was applied to reduce the risk of Type I error from multiple testing. Subgroup analyses with small sample sizes were interpreted as exploratory.

Analyses were conducted using R (v4.3.0) and SPSS (v28.0), with two-sided p<0.05 considered statistically significant for the primary analyses.

## 3 Results

### 3.1 Propensity matching and baseline characteristics

The study involved adult patients aged 18 and older who had undergone percutaneous coronary intervention (PCI) with at least one Drug-Eluting Stent (DES) and completed follow-up coronary angiography between May 2019 and December 2024. Exclusions included a history of CABG, acute STEMI or cardiogenic shock during follow-up, incomplete data for metabolic score calculations, and severe comorbidities like advanced hepatic or renal dysfunction or active malignancy. The initial cohort of 1,650 patients was reduced to 774 (387 matched pairs) after propensity score matching (PSM). (**Figure 1**) Propensity-score matching yielded 387 matched ISR–control pairs (N = 774). Most prespecified covariates achieved acceptable balance after matching; a small residual imbalance remained for vessel size. Eight of nine covariates achieved an absolute standardized mean difference <0.10 after matching. Stent length, despite a nominal post-matching between-group p-value in the original table, showed adequate balance by SMD (|SMD|=0.091). Reference vessel size showed a small residual imbalance (|SMD|=0.111), although this represented a marked reduction from its pre-matching value of 0.892. The maximum absolute SMD decreased from 0.892 before matching to 0.111 after matching. Propensity scores demonstrated substantial overlap between groups, with an empirical common-support interval of 0.0069–0.9590. Sixty-five ISR patients (7.9%) and six controls (0.7%) lay outside this interval. All 387 matched pairs satisfied the prespecified 0.062 caliper, with a maximum within-pair propensity-score difference of 0.062 (0.20 SD, corresponding to approximately 0.062 on the raw scale). Patients in the ISR group showed significantly higher median values for the TyG index (9.14 vs. 8.97), AIP (0.57 vs. 0.46), and METS-IR (42.2 vs. 41.9) compared to controls. (all p < 0.05) (**Table 1**).

**Figure 1:**
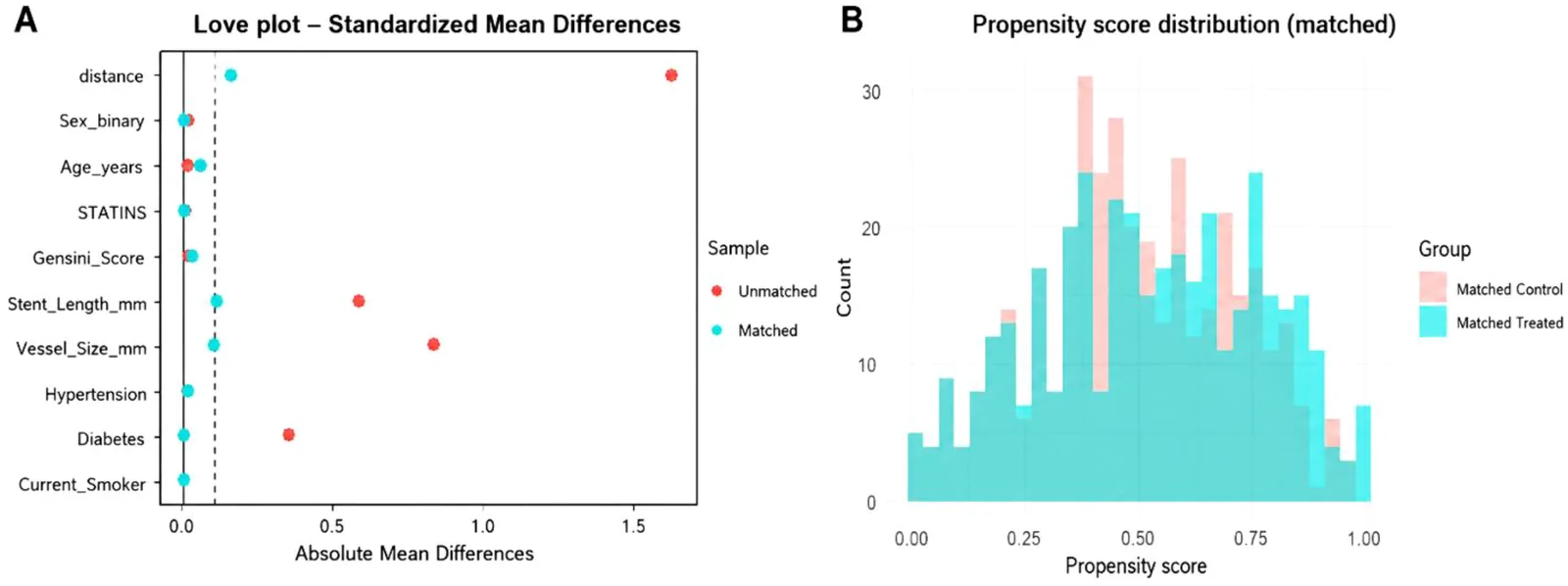
Propensity score matching covariate balance. Evaluation of covariate balance before and after matching. **(A)** Love plot of Standardized Mean Differences (SMD) comparing the unmatched (red) and matched (blue) samples. Following 1:1 nearest-neighbor propensity score matching, 8/9 covariates <0.10; vessel size showed minor residual imbalance (SMD 0.111). **(B)**. Propensity score distribution demonstrating substantial and symmetric overlaps between the matched treated (ISR) and control (No ISR) groups, confirming the robust comparability of the analytic cohort.

**Table 1:** Baseline characteristics stratified by ISR severity (before and after PSM).

Variable	Before PSM				After PSM			
	No ISR (n=830)	Less severe (n=451)	Very severe (n=369)	P	No ISR (n=387)	Less severe (n=201)	Very severe (n=186)	P
Demographics								
Age (years)	64.87 ± 9.06	64.72 ± 8.84	64.72 ± 9.17	0.946	64.82 ± 9.05	65.62 ± 9.08	64.78 ± 8.78	0.547
Sex (Male)	593 (71.4%)	325 (72.1%)	276 (74.8%)	0.481	271 (70.0%)	139 (69.2%)	132 (71.0%)	0.927
Anthropometrics								
BMI (kg/m^2^)	26.30 [23.8–29.0]	26.50 [23.8–29.1]	26.70 [23.8–29.4]	0.619	26.71 ± 3.81	26.60 ± 3.69	26.38 ± 3.82	0.616
Comorbidities & Risk Factors								
Hypertension	567 (68.3%)	302 (67.0%)	252 (68.3%)	0.873	268 (69.3%)	136 (67.7%)	127 (68.3%)	0.920
Diabetes	295 (35.5%)	323 (71.6%)	252 (68.3%)	<0.001	222 (57.4%)	119 (59.2%)	103 (55.4%)	0.749
Current Smoker	328 (39.5%)	185 (41.0%)	136 (36.9%)	0.473	137 (35.4%)	72 (35.8%)	65 (34.9%)	0.984
Medications								
Chinese Drug	276 (33.3%)	121 (26.8%)	95 (25.7%)	0.009	125 (32.3%)	56 (27.9%)	48 (25.8%)	0.231
β-Blocker	599 (72.2%)	324 (71.8%)	268 (72.6%)	0.969	265 (68.5%)	132 (65.7%)	131 (70.4%)	0.597
Statins	800 (96.4%)	444 (98.4%)	360 (97.6%)	0.091	374 (96.6%)	195 (97.0%)	181 (97.3%)	0.905
Fibrates	88 (10.6%)	22 (4.9%)	9 (2.4%)	<0.001	31 (8.0%)	10 (5.0%)	3 (1.6%)	0.007
Presenting symptoms								
Chest pain	0 (0.0%)	232 (51.4%)	189 (51.2%)	<0.001	0 (0.0%)	101 (50.2%)	98 (52.7%)	<0.001
PAL	0 (0.0%)	127 (28.2%)	110 (29.8%)	<0.001	0 (0.0%)	54 (26.9%)	56 (30.1%)	<0.001
Others	16 (1.9%)	42 (9.3%)	37 (10.0%)	<0.001	6 (1.6%)	21 (10.4%)	15 (8.1%)	<0.001
Routine FU	814 (98.1%)	29 (6.4%)	22 (6.0%)	<0.001	381 (98.4%)	13 (6.5%)	10 (5.4%)	<0.001
Presentation category: Symptoms driven	16 (1.9%)	422 (93.6%)	347 (94.0%)	<0.001	6 (1.6%)	188 (93.5%)	176 (94.6%)	<0.001
Presentation category: Routine FU	814 (98.1%)	29 (6.4%)	22 (6.0%)	<0.001	381 (98.4%)	13 (6.5%)	10 (5.4%)	<0.001
Laboratory – Metabolic								
Glucose (mg/dL)	115.75 [99.3–135.6]	133.30 [117.4–146.7]	131.60 [113.7–145.5]	<0.001	126.80 [108.1–142.8]	129.10 [112.6–141.6]	123.40 [108.0–145.3]	0.632
Triglycerides (mg/dL)	129.65 [98.1–175.1]	143.70 [103.6–197.2]	155.20 [108.6–203.8]	<0.001	129.00 [94.3–170.9]	141.10 [103.7–190.2]	160.95 [117.2–212.0]	<0.001
HDL-C (mg/dL)	45.90 [38.9–52.2]	44.30 [36.9–51.1]	42.80 [35.5–50.6]	<0.001	45.90 [38.6–51.3]	43.60 [37.2–50.3]	42.90 [36.1–50.9]	0.018
TyG Index	8.92 [8.6–9.2]	9.13 [8.8–9.5]	9.20 [8.8–9.5]	<0.001	8.97 [8.6–9.3]	9.09 [8.8–9.4]	9.18 [8.8–9.5]	<0.001
AIP	0.46 [0.3–0.6]	0.53 [0.4–0.7]	0.59 [0.4–0.7]	<0.001	0.46 [0.3–0.6]	0.54 [0.4–0.7]	0.60 [0.5–0.7]	<0.001
METS-IR	41.09 [36.8–45.6]	42.43 [37.5–47.3]	43.04 [38.1–48.0]	<0.001	41.92 [38.0–46.2]	42.62 [37.9–47.7]	41.85 [38.1–46.8]	0.561
Laboratory – Blood Count								
WBC (×10^9^/L)	6.17 [5.2–7.5]	6.30 [5.2–7.4]	6.10 [5.0–7.6]	0.603	6.10 [5.1–7.3]	6.30 [5.3–7.3]	6.08 [4.9–7.5]	0.413
RBC (×10^12^/L)	4.4 [4.0–4.7]	4.4 [4.0–4.8]	4.4 [4.0–4.7]	0.921	4.4 [4.0–4.7]	4.5 [4.1–4.8]	4.4 [4.1–4.7]	0.306
HGB (g/L)	134.9 [122–145]	134.0 [122–145]	135.0 [123–145]	0.509	134.0 [122–145]	136.0 [124–146]	135.0 [124–144]	0.686
HCT (%)	40.5 [37.0–43.5]	40.6 [36.9–43.4]	40.6 [37.4–43.6]	0.48	40.6 [36.9–43.4]	40.8 [37.2–43.9]	40.6 [37.5–43.6]	0.624
PLT (×10^9^/L)	194.0 [160–235]	193.0 [160–233]	194.0 [159–233]	0.847	194.0 [159–234]	188.0 [156–231]	194.0 [163–232]	0.315
NEU (×10^9^/L)	4.0 [3.2–5.0]	4.0 [3.2–5.0]	3.9 [3.1–5.0]	0.730	3.9 [3.1–4.8]	4.0 [3.3–5.0]	3.9 [3.1–5.0]	0.389
LYM (×10^9^/L)	1.4 [1.1–1.9]	1.4 [1.1–1.9]	1.4 [1.0–1.8]	0.501	1.4 [1.1–1.8]	1.4 [1.0–1.8]	1.4 [1.0–1.8]	0.470
MO (×10^9^/L)	0.5 [0.4–0.6]	0.5 [0.4–0.6]	0.5 [0.4–0.6]	0.915	0.5 [0.4–0.6]	0.5 [0.4–0.6]	0.5 [0.4–0.6]	0.730
EOS (×10^9^/L)	0.1 [0.1–0.2]	0.1 [0.1–0.2]	0.1 [0.1–0.2]	0.694	0.1 [0.1–0.2]	0.1 [0.1–0.2]	0.1 [0.1–0.2]	0.745
Angiographic/Lesion Characteristics								
Vessel Size (mm)	2.98 [2.7–3.2]	2.46 [2.2–2.9]	2.46 [2.3–2.9]	<0.001	2.81 [2.6–3.1]	2.81 [2.4–3.2]	2.65 [2.3–3.2]	0.070
Stent Length (mm)	25.70 [20.0–31.6]	32.50 [25.2–37.0]	32.50 [25.7–38.0]	<0.001	27.70 [23.2–34.6]	31.50 [23.5–35.5]	31.30 [24.2–37.2]	0.022
Gensini Score	40.11 ± 12.29	40.37 ± 12.36	40.19 ± 12.62	0.940	40.47 ± 12.00	40.75 ± 13.33	40.84 ± 12.65	0.936
QCA % Diameter Stenosis	29.2 [20.1–37.5]	56.8 [53.3–61.0]	87.2 [81.1–93.5]	<0.001	28.7 [19.9–35.8]	56.7 [53.4–60.8]	87.2 [82.5–93.9]	<0.001
QCA Lesion Length (mm)	6.6 [4.8–8.3]	10.0 [7.3–12.4]	42.6 [29.5–51.7]	<0.001	6.4 [4.6–8.2]	10.3 [7.3–12.7]	42.7 [29.4–50.7]	<0.001
QCA Min Luminal Dia (mm)	2.1 [1.8–2.4]	1.1 [0.9–1.3]	0.3 [0.3–0.5]	<0.001	2.0 [1.8–2.3]	1.2 [1.0–1.4]	0.3 [0.3–0.5]	<0.001
Bifurcation Lesion	212 (25.5%)	97 (21.5%)	89 (24.1%)	0.273	103 (26.6%)	44 (21.9%)	44 (23.7%)	0.422
Ostial Lesion	149 (18.0%)	72 (16.0%)	56 (15.2%)	0.425				

Statistical Methods: Normality was tested using the Shapiro–Wilk test. Data presented as Mean ± SD (compared via ANOVA) or Median [IQR] (compared via Kruskal–Wallis test) for continuous variables. Categorical variables are presented as n (%) and compared using the Chi-square test.

### 3.2 Correlation among major predictors

A correlation matrix of key clinical, metabolic, and procedural variables is shown in **Figure 2**. The metabolic indices demonstrated strong intercorrelation: TyG with AIP (r = 0.78), TyG with METS-IR (r = 0.72), and AIP with METS-IR (r = 0.62). Vessel size showed a moderate inverse correlation with percent diameter stenosis (r = -0.35). Low correlations (<0.25) were observed between metabolic and procedural metrics, confirming their analytical independence in subsequent regression models (**Figure 2**).

**Figure 2:**
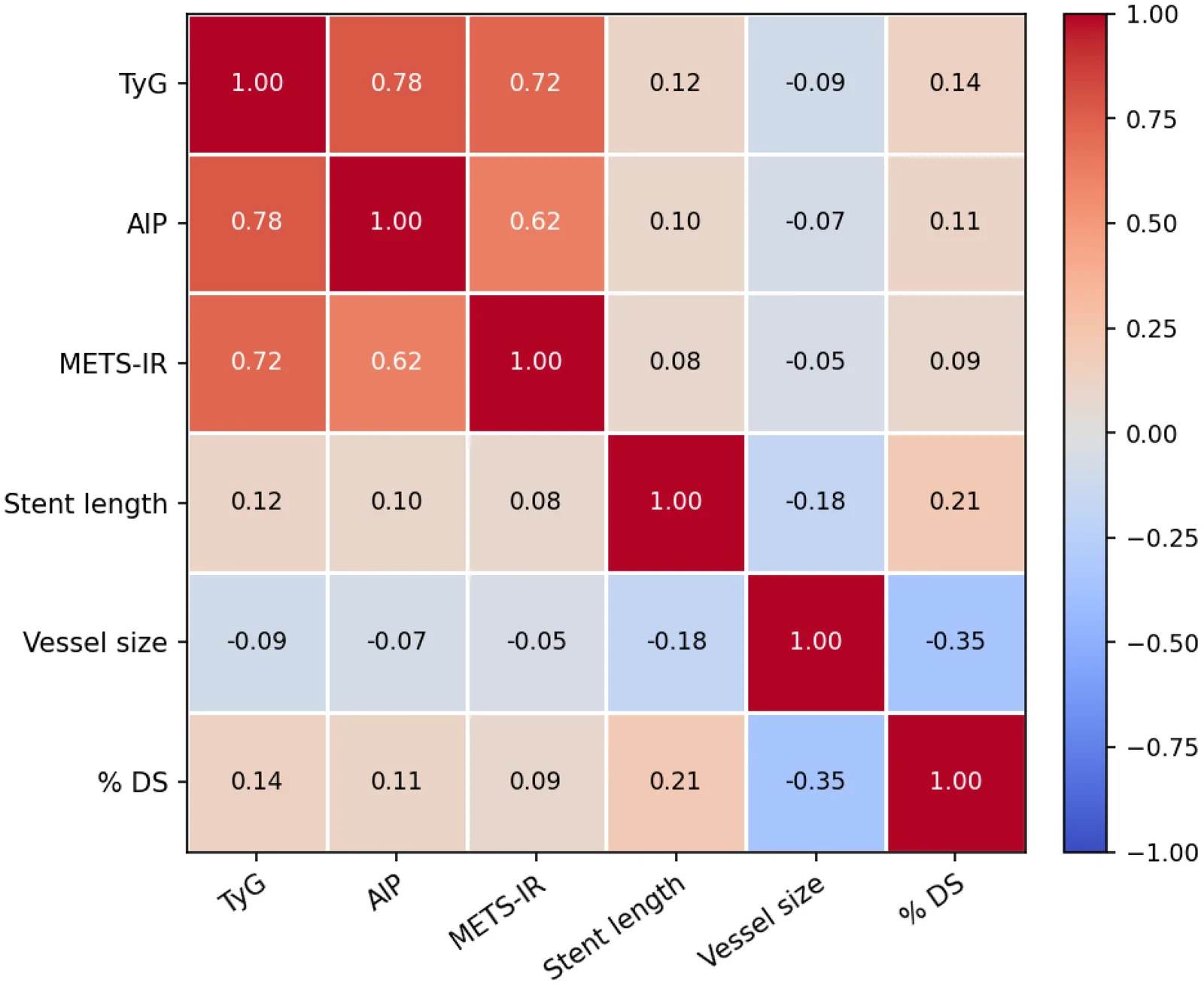
Correlation matrix among metabolic and procedural variables. strong correlation (r=0.62–0.78) between TyG, AIP, and METS-IR, justifying separate-model analyses.

### 3.3 Determinants of angiographic severity and timing

Among the 387 matched ISR cases, metabolic indices scaled with angiographic severity: TyG exhibited a graded increase from 8.97 [IQR: 8.6–9.3] in controls to 9.09 [8.8–9.4] in less severe ISR, peaking at 9.18 [8.8–9.5] in very severe cases (p<0.001) (**Table 2**). For the association with very severe ISR, AIP provided the greatest discriminative improvement over the baseline model (AUC 0.682), reclassifying 22.1% of patients (NRI = 0.324). Despite their Higher baseline levels, none of these indices were significantly associated with early angiographic detection (≤12 months) (**Table 2**). No significant correlation was observed between TyG (r = 0.04, p = 0.42), AIP (r = 0.02, p = 0.68), or METS-IR (r = 0.01, p = 0.81) and the continuous time to angiographic detection.

**Table 2:** Baseline characteristics stratified by early vs. late ISR cases.

Variable	Categories		
	Early detection ISR (n=246)	Late detection ISR (n=574)	P
Demographics			
Age (years)	65.0 [58–71]	65.0 [59–71]	0.463
Sex (Male)	178 (72.4%)	423 (73.7%)	0.757
Anthropometrics			
BMI (kg/m^2^)	26.7 [23.7–29.6]	26.5 [23.8–29.1]	0.922
Comorbidities & Risk Factors			
Hypertension	166 (67.5%)	388 (67.6%)	1.000
Diabetes	169 (68.7%)	406 (70.7%)	0.617
Smoker	94 (38.2%)	227 (39.5%)	0.779
Medications			
Chinese Drug	171 (69.5%)	433 (75.4%)	0.093
β-Blocker	177 (72.0%)	415 (72.3%)	0.986
Statins	243 (98.8%)	561 (97.7%)	0.474
Fibrates	9 (3.7%)	22 (3.8%)	1.000
Clinical Presentation			
Chest pain	121 (49.2%)	182 (49.3%)	0.421
PAL (Palpitations)	77 (31.3%)	110 (29.8%)	0.334
Others	20 (8.1%)	36 (9.8%)	0.367
Category of C/C			
Symptoms	229 (93.1%)	540 (94.1%)	0.603
Routine follow-up	17 (6.9%)	34 (5.9%)	—
Laboratory – Metabolic			
Fasting Glucose (mg/dL)	130.4 [114–143]	133.3 [117–147]	0.389
Triglycerides (mg/dL)	155.2 [114–206]	144.0 [103–198]	0.164
HDL-C (mg/dL)	44.7 [37–52]	43.1 [36–51]	0.037
TyG Index	9.2 [8.8–9.5]	9.1 [8.8–9.5]	0.180
AIP	0.58 [0.39–0.68]	0.55 [0.38–0.70]	0.844
METS-IR	42.7 [37.5–47.4]	42.5 [37.9–47.7]	0.672
Laboratory – Blood Count			
WBC (×10^9^/L)	6.3 [5.2–7.6]	6.1 [5.1–7.5]	0.441
RBC (×10^12^/L)	4.4 [4.0–4.7]	4.4 [4.0–4.7]	0.547
HGB (g/L)	135 [121–146]	135 [123–145]	0.485
HCT (%)	40.3 [36.7–43.3]	40.6 [37.3–43.6]	0.288
PLT (×10^9^/L)	196 [160–231]	192 [159–234]	0.601
NEU (×10^9^/L)	4.0 [3.1–5.1]	3.9 [3.2–5.0]	0.391
LYM (×10^9^/L)	1.4 [1.1–1.9]	1.4 [1.0–1.8]	0.647
MO (×10^9^/L)	0.5 [0.4–0.6]	0.5 [0.4–0.6]	0.678
EOS (×10^9^/L)	0.1 [0.1–0.2]	0.1 [0.1–0.2]	0.496
Angiographic/Lesion Characteristics			
Vessel Size (mm)	2.5 [2.3–2.9]	2.5 [2.2–2.9]	0.273
Stent Length (mm)	31.9 [25.3–37.7]	32.7 [25.3–37.5]	0.611
Gensini Score	39.6 [30.9–47.9]	40.3 [31.3–49.8]	0.366
QCA % Stenosis	63.9 [55.9–87.0]	63.4 [55.9–85.5]	0.688
QCA Lesion Length (mm)	14.3 [9.7–39.9]	13.7 [9.3–40.8]	0.867
QCA Min Luminal Dia (mm)	0.8 [0.3–1.2]	0.8 [0.4–1.1]	0.821
Bifurcation Lesion	56 (22.8%)	130 (22.6%)	1.000
Ostial Lesion	31 (12.6%)	97 (16.9%)	0.147

Baseline demographic, clinical, metabolic, and procedural characteristics stratified by early versus late angiographic detection of in-stent restenosis (ISR). Data is presented for both the original unmatched cohort (Before PSM). Continuous variables are expressed as Median [IQR] and compared via the Mann-Whitney U test. Categorical variables are expressed as n (%) and compared via the Chi-square test.

For severity, none of the indices significantly improved AUC according to paired DeLong testing. AIP showed significant NRI and IDI estimates, but because ΔAUC was small and its confidence interval included zero, this should be described as limited reclassification improvement rather than a clear improvement in overall discrimination.

### 3.4 Association with ISR occurrence

Evaluating indices sequentially, the baseline clinical model yielded an AUC of 0.716. The TyG index significantly improved model discrimination (AUC 0.756; ΔAUC +0.040) and reclassified 25.3% of patients (NRI = 0.279, IDI = 0.032). AIP similarly improved the AUC to 0.766 (ΔAUC +0.050) with an NRI of 0.393. METS-IR did not yield a clinically meaningful improvement (AUC 0.716; NRI = -0.005) (**Table 3**, **Figure 3**). All covariate ORs with their 95% CI are available in the ANNEX.

**Table 3:** Incremental discriminative and reclassification performance of metabolic indices for ISR occurrence and severity.

Outcome	Model	AUC (95% CI)	ΔAUC (95% CI)	DeLong P	NRI (95% CI)	NRI P	IDI (95% CI)	IDI P
ISR Occurrence	Baseline model	0.716 (0.672–0.763)	—	—	—	—	—	—
	+ TyG	0.756 (0.708–0.803)	+0.040 (0.017–0.092)	0.003	0.279 (0.145–0.429)	0.002	0.032 (0.011–0.058)	0.002
	+ AIP	0.772 (0.726–0.806)	+0.056 (0.031–0.116)	<0.001	0.393 (0.238–0.532)	0.002	0.042 (0.017–0.075)	0.002
	+ METS-IR	0.716 (0.671–0.748)	+0.00 (−0.004–0.032)	0.642	0.067 (−0.067–0.202)	0.402	0.003 (0.000–0.017)	0.004
ISR Severity	Baseline model	0.658 (0.591–0.735)	—	—	—	—	—	—
	+ TyG	0.676 (0.619–0.743)	+0.018 (−0.003–0.059)	0.431	0.161 (−0.056–0.365)	0.166	0.010 (0.000–0.038)	0.010
	+ AIP	0.682 (0.622–0.752)	+0.026 (−0.001–0.068)	0.295	0.324 (0.068–0.515)	0.030	0.014 (0.001–0.047)	0.008
	+ METS-IR	0.657 (0.590–0.735)	−0.001 (−0.004–0.025)	0.818	−0.059 (−0.162–0.288)	0.675	0.000 (0.000–0.013)	0.036

The analysis of in-stent restenosis (ISR) included 774 matched patients (387 ISR cases and 387 controls). Severity analysis focused on the 387 ISR patients. The baseline model accounted for age, sex, hypertension, diabetes, smoking status, statin use, stent length, and vessel size. Area Under the Curve (AUC) values and 95% confidence intervals (CIs) were reported, with the change in AUC (ΔAUC) indicating differences from the baseline. Paired DeLong tests compared correlated ROC curves, and confidence intervals for ΔAUC, continuous Net Reclassification Improvement (NRI), and Integrated Discrimination Improvement (IDI) were calculated via bootstrap resampling. Improvement in reclassification and discrimination was indicated by values of RI and IDI greater than 0.

**Figure 3:**
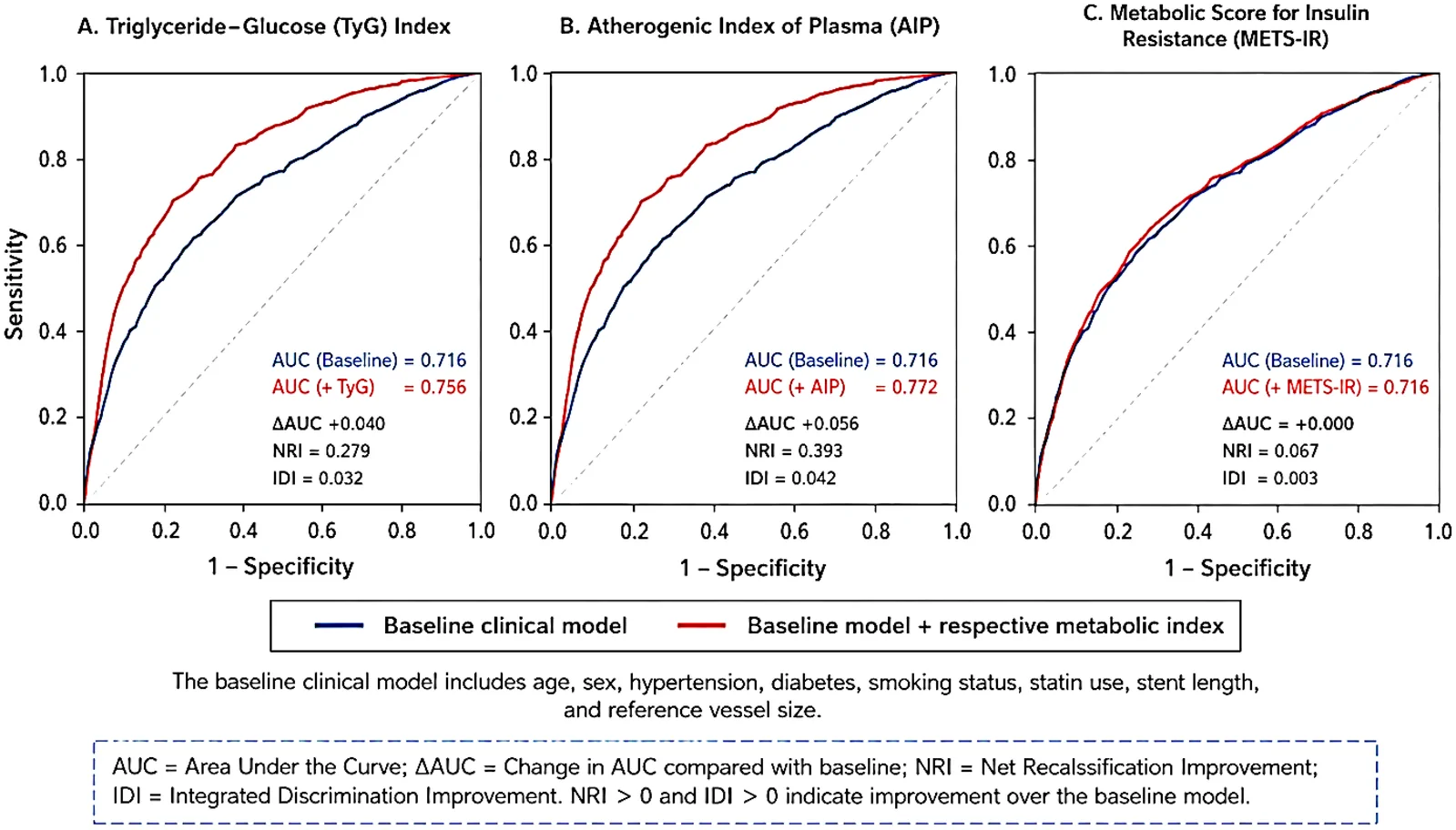
Receiver operating characteristic curves for prediction of angiographically detectedin-stent restenosis in the propensity-score-matched cohort. **(A)** Baseline clinical model (blue line) compared with the model additionally incorporating the Triglyceride-Glucose (TyG) index (orange line). The baseline model included age, sex, hypertension, diabetes mellitus, smoking status, statin use, stent length, and vessel size. The baseline model had an AUC of 0.716 (95% CI 0.672–0.763); addition of TyG yielded an AUC of 0.756 (95% CI 0.708–0.803; ΔAUC +0.040; paired DeLong p=0.003; NRI=0.279; IDI=0.032). **(B)** Baseline clinical model (blue line) compared with the model additionally incorporating the Atherogenic Index of Plasma (AIP) (red line). Addition of AIP yielded an AUC of 0.772 (95% CI 0.726–0.806; ΔAUC +0.056; p<0.001; NRI=0.393; IDI=0.042). **(C)** Baseline clinical model (blue line) compared with the model additionally incorporating the Metabolic Score for Insulin Resistance (METS-IR) (green line). Addition of METS-IR yielded an AUC of 0.716 (95% CI 0.671–0.748; ΔAUC approximately 0.000; p=0.642; NRI=0.067; IDI=0.003), with no meaningful improvement in discrimination. Analyses were performed in the matched cohort (N=774; 387 ISR cases and 387 controls). The diagonal dashed line represents chance discrimination (AUC=0.50).

A very useful interpretation is that TyG and AIP significantly improved discrimination for ISR occurrence, whereas METS-IR did not materially improve AUC or NRI. Although the METS-IR IDI p-value was statistically significant, the absolute IDI was only 0.003 and its AUC and NRI results were null; therefore, this should not be presented as meaningful incremental discrimination.

We additionally performed 1,000 matched-pair bootstrap resamples. The bootstrap 95% CI for ΔAUC excluded zero for TyG (0.016–0.086) and AIP (0.030–0.109), but not for METS-IR (−0.006 to 0.028). Although the direct DeLong comparison suggested a modest numerical advantage of AIP over TyG (ΔAUC=0.023; p=0.032), its matched-pair bootstrap 95% CI included zero (−0.002 to 0.040); see Annex.

### 3.5 Subgroup analysis by sex

Baseline clinical variables were inherently stronger at discriminating severe ISR in women (AUC = 0.734) compared to men (AUC = 0.615). Although men exhibited higher absolute baseline levels of AIP, women developed very severe restenosis at lower metabolic thresholds (optimal AIP cutoff 0.457 for women vs 0.547 for men). Furthermore, elevated AIP acted as a risk amplifier in the female cohort, associated with a six-fold increase in the odds of Very Severe ISR (OR 6.19, p=0.046). Conversely, the introduction of metabolic indices increased overall discriminative power more broadly in the male cohort (AIP ΔAUC +0.022; TyG ΔAUC +0.033) than in the female cohort (AIP ΔAUC +0.037; TyG ΔAUC +0.027) (**Figure 4**).

**Figure 4:**
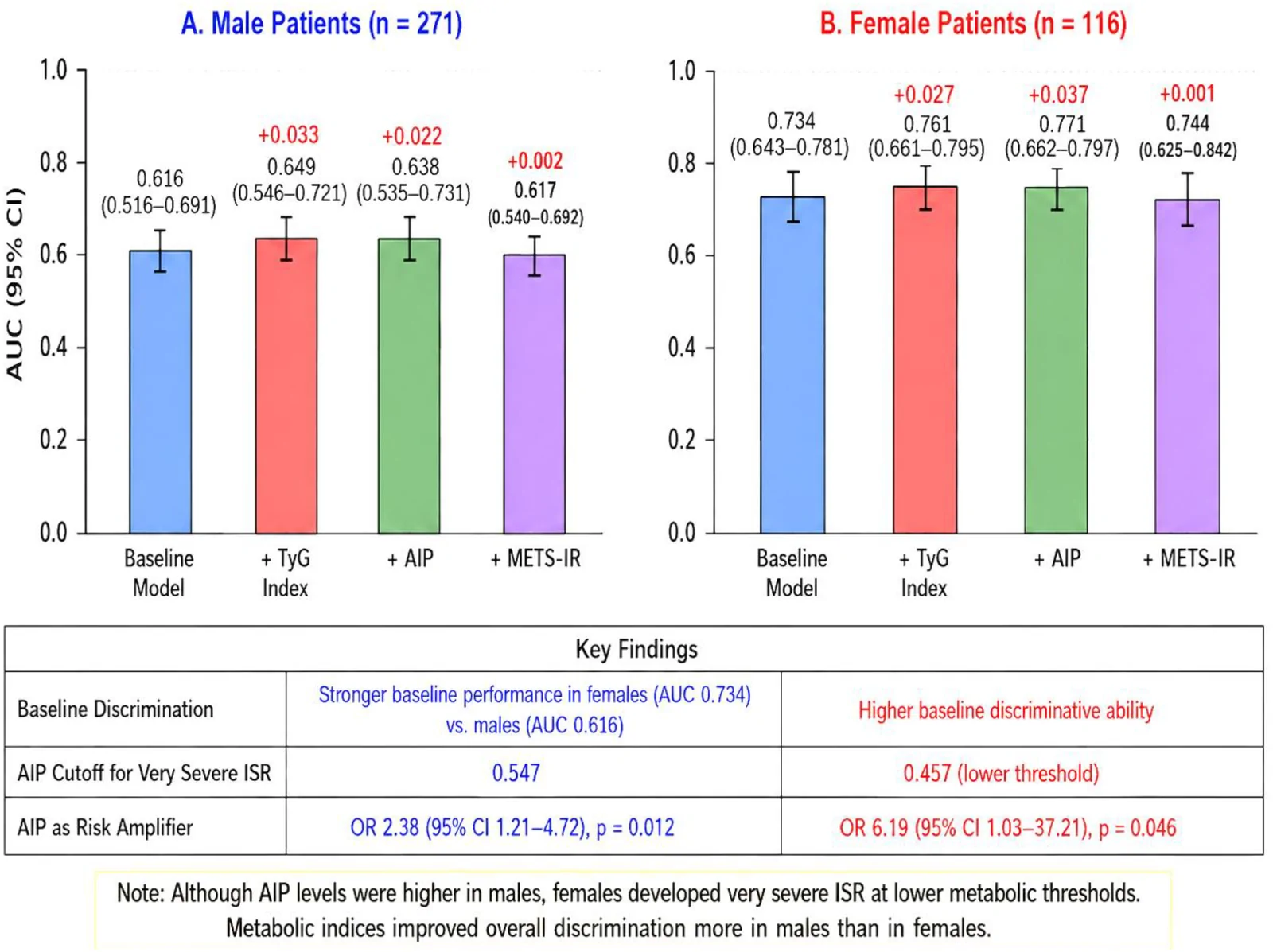
Sex-stratified receiver operating characteristic curves for discrimination of very severe versus less severe in-stent restenosis among matched ISR cases. **(A)** Female cohort (n=116). The baseline clinical model (blue line) yielded an AUC of 0.734 (95% CI 0.643–0.781); addition of TyG (orange line) increased the AUC to 0.761 (95% CI 0.661–0.795; ΔAUC +0.027; paired DeLong p=0.184); addition of AIP (red line) increased the AUC to 0.771 (95% CI 0.662–0.797; ΔAUC +0.037; p=0.052). **(B)** Male cohort (n=271). The baseline clinical model (blue line) yielded an AUC of 0.616 (95% CI 0.516–0.691); addition of TyG (orange line) yielded an AUC of 0.649 (95% CI 0.546–0.721; ΔAUC +0.033; p=0.198); addition of AIP (red line) yielded an AUC of 0.638 (95% CI 0.535–0.731; ΔAUC +0.022; p=0.317). Models were adjusted for age, hypertension, diabetes mellitus, smoking status, statin use, stent length, and vessel size. These subgroup findings should be interpreted as exploratory. The diagonal dashed line represents chance discrimination (AUC=0.50).

Logistic regression models were developed to compare a baseline clinical model with one enhanced by individual metabolic indices for ISR detection timing, stratified by sex. The Baseline Model included age, hypertension, diabetes, smoking status, statin use, stent length, and reference vessel size. In male patients, the baseline model had nearly random discriminative capability for early vs. late ISR timing, with minimal improvement from metabolic indices. In female patients, the models showed better baseline performance, but adding metabolic indices also provided limited improvement (**Table 4**).

**Table 4:** Sex-stratified discriminative performance for ISR severity (very severe vs. less severe).

Outcome	Sex	N	Baseline AUC (95% CI)	+TyG AUC (95% CI)	ΔAUC	DeLong P	+AIP AUC (95% CI)	ΔAUC	DeLong P
ISR Severity	Female	116	0.734 (0.643–0.781)	0.761(0.661–0.795)	+0.027	0.184	0.771 (0.662–0.797)	+0.037	0.052
	Male	271	0.616 (0.516–0.691)	0.649 (0.546–0.721)	+0.033	0.198	0.638 (0.535–0.731)	+0.022	0.317
Timing of ISR Detection	Female	116	0.705 (0.601–0.740)	0.719 (0.607–0.724)	+0.014	0.492	0.716 (0.612–0.727)	+0.011	0.469
	Male	271	0.636 (0.615–0.686)	0.635 (0.624–0.705)	−0.001	0.671	0.630 (0.626–0.707)	−0.006	0.688

Models adjusted for age, hypertension, diabetes, smoking, statin use, stent length, and vessel size.

However, formal sex × index interaction testing for ISR severity showed no evidence of effect modification: TyG p=0.630, AIP p=0.503. Therefore, differences between male and female severity subgroup estimates are considered exploratory and are not interpreted as evidence of sex-specific susceptibility.

### 3.6 Subgroup analysis by clinical presentation

Patients were stratified by their categorized reason for presentation into symptomatic (n=364) and routine follow-up (n=23). The discriminative benefit of TyG and AIP was driven entirely by patients with systemic, non-acute presentations such as generalized fatigue (TyG ΔAUC +0.013) (**Figure 5**). Conversely, the metabolic indices provided no additive discriminative value over baseline variables in patients presenting with acute, localized symptoms such as chest pain (TyG ΔAUC -0.001) or palpitations (TyG ΔAUC -0.003) (**Table 5**). We interpreted the clinical presentation analyses as sensitivity and exploratory analyses because of the pronounced imbalance in the reason for follow-up angiography.

**Figure 5:**
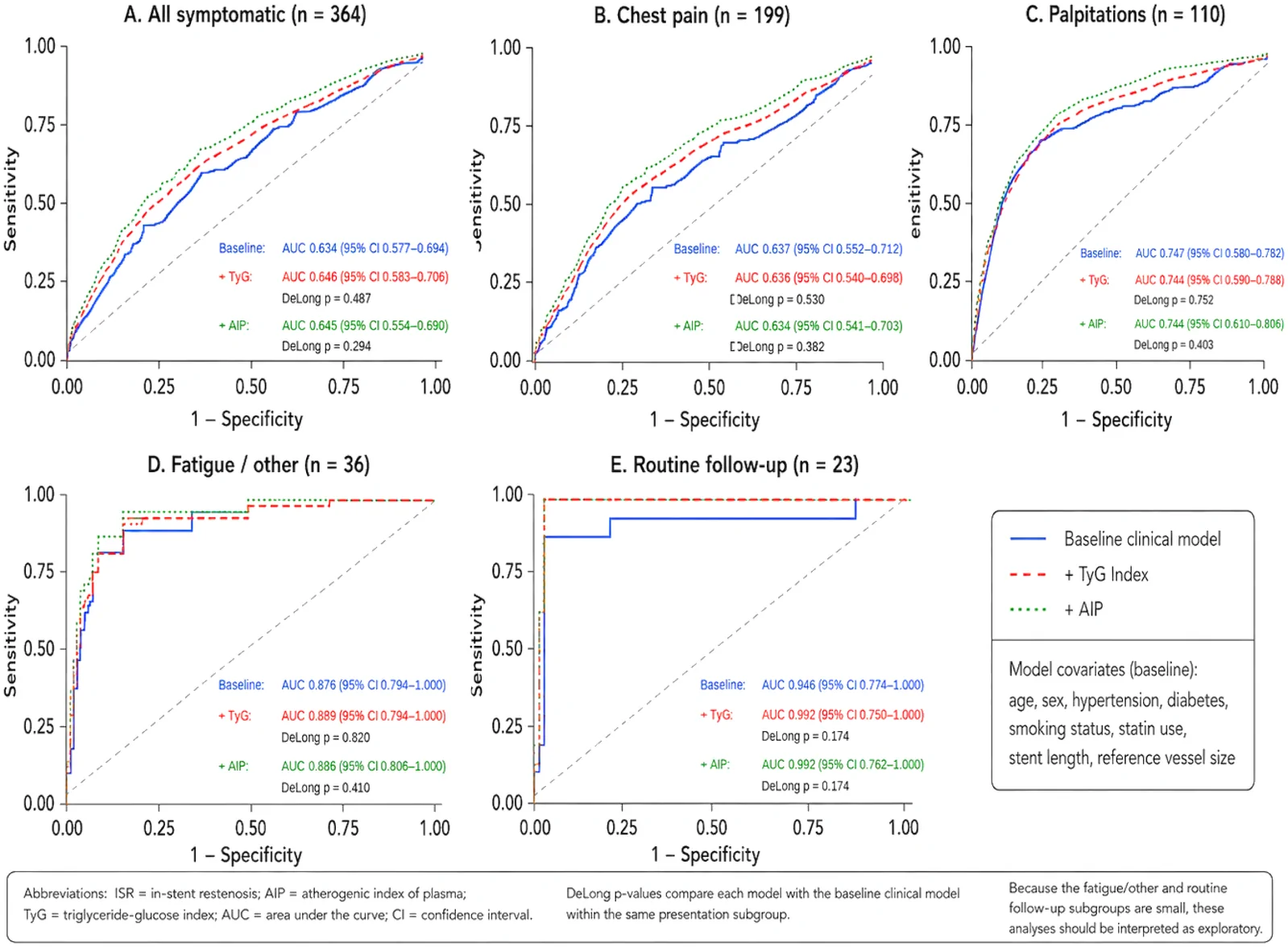
Receiver operating characteristic curves for discrimination of very severe versus less severe in-stent restenosis across clinical presentation subgroups. **(A)** All symptomatic patients (n=364). Baseline AUC: 0.634 (95% CI 0.577–0.694); +TyG AUC: 0.646 (95% CI 0.583–0.706; ΔAUC +0.013; p=0.487); +AIP AUC: 0.645 (95% CI 0.554–0.690; ΔAUC +0.012; p=0.294). **(B)** Chest pain subgroup (n=199). Baseline AUC: 0.637 (95% CI 0.552–0.712); +TyG AUC: 0.636 (95% CI 0.540–0.698; ΔAUC −0.001; p=0.530); +AIP AUC: 0.634 (95% CI 0.541–0.703; ΔAUC −0.003; p=0.382). **(C)** Palpitations subgroup (n=110). Baseline AUC: 0.747 (95% CI 0.580–0.782); +TyG AUC: 0.744 (95% CI 0.589–0.788; ΔAUC −0.003; p=0.752); +AIP AUC: 0.744 (95% CI 0.610–0.806; ΔAUC −0.003; p=0.403). **(D)** Fatigue/other presentations subgroup (n=36). Baseline AUC: 0.876 (95% CI 0.794–1.000); +TyG AUC: 0.889 (95% CI 0.794–1.000; ΔAUC +0.013; p=0.820); +AIP AUC: 0.886 (95% CI 0.806–1.000; ΔAUC +0.010; p=0.410). **(E)** Routine follow-up subgroup (n=23). Baseline AUC: 0.946 (95% CI 0.774–1.000); +TyG AUC: 0.992 (95% CI 0.750–1.000; ΔAUC +0.046; p=0.174); +AIP AUC: 0.992 (95% CI 0.762–1.000; ΔAUC +0.046; p=0.174). The blue line represents the baseline clinical model, the orange line represents the model with TyG, and the red line represents the model with AIP. Models were adjusted for age, hypertension, diabetes mellitus, smoking status, statin use, stent length, and vessel size. The fatigue/other and routine follow-up analyses should be considered exploratory because of the small subgroup sizes. The diagonal dashed line represents chance discrimination (AUC=0.50).

**Table 5:** Subgroup analysis by clinical presentation.

Clinical Presentation	N	Baseline AUC (95% CI)	+TyG AUC (95% CI)	ΔAUC	DeLong P	+AIP AUC (95% CI)	ΔAUC	DeLong P
All symptomatic	364	0.634 (0.577–0.694)	0.646 (0.583–0.706)	+0.013	0.487	0.645 (0.554–0.690)	+0.012	0.294
Chest pain	199	0.637 (0.552–0.712)	0.636 (0.540–0.698)	-0.001	0.530	0.634 (0.541–0.703)	-0.003	0.382
Palpitations	110	0.747 (0.580–0.782)	0.744 (0.589–0.788)	-0.003	0.752	0.744 (0.610–0.806)	-0.003	0.403
Fatigue/other	36	0.876(0.794–1.000)	0.889 (0.794–1.000)	0.013	0.820	0.886 (0.806–1.000)	+0.01	0.410
Routine follow-up	23	0.946 (0.774–1.000)	0.992 (0.750–1.000)	+0.046	0.174	0.992 (0.762–1.000)	+0.046	0.174

Larger numerical AUC differences were observed in the small fatigue/routine-follow-up subgroups, but none was significant.

To assess the impact of this imbalance, a sensitivity analysis was restricted to patients undergoing routine follow-up. Among 865 such patients, including 51 ISR cases and 814 controls, TyG was not significantly associated with ISR (OR 1.34, 95% CI 0.67–2.72; p=0.405), nor was AIP (OR 2.34, 95% CI 0.48–11.80; p=0.295) or METS-IR (OR 1.00, 95% CI 0.95–1.04; p=0.893).

Within the symptomatic group, TyG (OR 5.47, 95% CI 1.84–17.25; p=0.002) and AIP (OR 31.34, 95% CI 3.33–316.45; p=0.003) showed significant associations, whereas METS-IR did not. However, this subgroup contained only 16 controls, resulting in wide confidence intervals and limited estimate stability. These results are therefore considered exploratory.

Because the routine-follow-up and fatigue/other subgroups were small, these high AUC estimates are considered potentially unstable and are not used to support clinical recommendations. Sample sizes and confidence intervals are provided with the subgroup ROC results to make the associated uncertainty explicit. Formal presentation × metabolic-index interaction testing for ISR severity did not demonstrate significant effect modification. The interaction p-values were 0.365 for TyG, 0.110 for AIP, and 0.096 for METS-IR; all remained nonsignificant after Benjamini-Hochberg correction.

Overall, the presentation analyses demonstrate substantial heterogeneity in angiographic ascertainment and support cautious interpretation of presentation-specific findings rather than claims of differential clinical utility.

## 4 Discussion

In this propensity-score-matched case-control study, we compared the associations of AIP, TyG, and METS-IR with angiographically detected ISR within the same cohort of patients undergoing follow-up coronary angiography after drug-eluting stent implantation. TyG and AIP improved discrimination beyond the baseline clinical and procedural model, whereas METS-IR provided little additional information. The baseline AUC of 0.716 increased to 0.756 after addition of TyG and to 0.772 after addition of AIP, while remaining essentially unchanged after addition of METS-IR. Matched-pair bootstrap analyses supported the incremental contribution of TyG and AIP, as the confidence intervals for ΔAUC excluded zero, whereas the corresponding interval for METS-IR did not. Although the direct DeLong comparison suggested a modest difference between AIP and TyG, the bootstrap confidence interval for this comparison included zero; therefore, these findings do not establish clear superiority of one index over the other. Previous studies have reported associations between TyG and ISR or adverse cardiovascular outcomes after PCI (13, 14). The present study extends these observations by comparing three metabolic indices within the same matched population and by examining their incremental discriminatory value against an identical clinical and procedural background. Recent studies have also underscored the value of clinical and metabolic risk markers in other PCI settings, including ST-segment resolution after primary PCI (15) and contrast-induced nephropathy in patients undergoing chronic total occlusion intervention (16). Although these outcomes differ from ISR, they provide additional support for evaluating simple metabolic indices in patients undergoing coronary intervention.

The stronger performance of TyG and AIP relative to METS-IR is biologically plausible but should be interpreted cautiously. AIP reflects the balance between triglycerides and HDL-C and has been associated with an atherogenic lipoprotein profile, whereas TyG integrates fasting triglyceride and glucose concentrations and is widely used as a surrogate marker of insulin resistance. Triglyceride-rich lipoproteins, impaired HDL function, insulin resistance, oxidative stress, and endothelial dysfunction have all been implicated in vascular injury and abnormal healing after coronary intervention (17, 18). By contrast, METS-IR incorporates BMI in addition to glucose and lipid variables and may reflect a broader metabolic phenotype. The limited value of METS-IR in this cohort may reflect the influence of the “obesity paradox” in post-PCI patients. By incorporating BMI, METS-IR may inadvertently capture protective aspects of adiposity—such as greater metabolic reserve and favorable adipokine profiles—that paradoxically improve outcomes in patients with established coronary disease. In contrast, TyG and AIP do not include BMI and may more directly reflect the lipid-glucose perturbations most relevant to neointimal hyperplasia and stent failure. Additionally, TyG and AIP may capture more dynamic metabolic states that are sensitive to periprocedural changes, whereas METS-IR, with its BMI component, reflects a more stable trait less responsive to the temporal metabolic fluctuations influencing ISR development. These observations do not diminish the clinical utility of METS-IR in other settings but highlight the importance of index selection in the specific context of post-stent vascular healing. The metabolic information captured by TyG and AIP may have been more closely related to angiographically detected ISR in this setting (19, 20). The results for ISR severity were less conclusive. Among the 387 matched patients with ISR, addition of TyG or AIP produced only modest increases in AUC, and neither improvement was statistically significant by paired DeLong testing. AIP showed favorable NRI and IDI estimates, but the small change in AUC and the confidence interval crossing zero argue against describing this as a clear improvement in overall severity discrimination. TyG likewise showed only borderline evidence of association with severity. The timing analyses were also negative: none of the three indices was significantly associated with early versus late ISR detection, and no meaningful correlation was observed with continuous time to angiographic detection. Because retrospective angiography identifies the time at which restenosis is detected rather than the time at which it develops biologically, these findings should not be interpreted as evidence regarding the rate of ISR progression.

The subgroup findings warrant particular caution. The baseline clinical model showed higher discrimination for severe ISR in women than in men, and the magnitude of the changes after adding TyG or AIP differed numerically between the two groups. However, formal sex-by-index interaction testing did not show significant effect modification. The observed sex differences should therefore be regarded as exploratory and should not be interpreted as evidence of differential biological susceptibility. Sex-related differences in coronary pathophysiology, lipid metabolism, vascular inflammation, and outcomes after PCI have been well described (21–23), but the present study was not sufficiently powered to establish whether the associations of metabolic indices with ISR differ materially between women and men. The analyses according to clinical presentation are similarly exploratory. The fatigue/other and routine-follow-up subgroups included only 36 and 23 patients, respectively, and the high AUC estimates observed in these groups are likely to be unstable. In addition, none of the presentation-specific additions of TyG or AIP significantly improved AUC, and formal presentation-by-index interaction testing was nonsignificant. These results therefore do not support a conclusion that the metabolic indices are preferentially useful in asymptomatic or atypically presenting patients. This is particularly important given the marked imbalance in angiographic indication between ISR cases and controls, which introduces the possibility of selection and ascertainment bias. ISR after contemporary DES implantation, remains a multifactorial process influenced by patient characteristics, lesion complexity, procedural factors, device characteristics, and subsequent vascular healing (24). Accordingly, the present findings are best interpreted as evidence of association and incremental discrimination within the studied angiographic cohort rather than as evidence of causation or prospective predictive performance.

### 4.1 Study limitations

This study has several limitations. First, the retrospective, single-center case-control design limits causal inference and generalizability. Because study inclusion depended on patients undergoing follow-up coronary angiography, the study cannot estimate the population incidence of ISR and remains susceptible to referral, selection, and ascertainment bias. This concern is particularly relevant to the substantial difference in angiographic indication between ISR cases and controls. Second, the metabolic indices were calculated from fasting measurements obtained during hospitalization for follow-up angiography and therefore reflect metabolic status at the time of angiographic assessment rather than exposure measured before or at the index PCI. Temporal and causal relationships between these indices and subsequent ISR development cannot therefore be established. Third, the timing analysis reflects the timing of angiographic detection rather than the biological onset of restenosis. Fourth, despite propensity-score matching and multivariable adjustment, residual confounding remains possible, particularly in relation to DES platform, intravascular imaging, procedural characteristics, medication intensity, and longitudinal treatment adherence. A small residual imbalance in vessel size also remained after matching. Fifth, the sex- and presentation-specific analyses involved relatively small subgroups and are therefore vulnerable to limited statistical power, unstable estimates, and model overfitting. Although the Benjamini-Hochberg procedure was applied to prespecified families of subgroup and interaction analyses, the number of secondary comparisons still warrants cautious interpretation. Finally, matched-pair bootstrap resampling was used to assess the robustness of incremental discrimination, but no independent external validation cohort was available. Prospective multicenter studies with metabolic measurements obtained before or at the time of PCI, standardized follow-up, detailed procedural characterization, and external validation are required before these indices can be considered for routine ISR risk stratification.

## 5 Conclusion

In this propensity-score-matched case-control study, TyG and AIP provided incremental discriminatory information for angiographically detected ISR beyond conventional clinical and procedural factors, whereas METS-IR offered little additional value. The evidence for ISR severity was less robust, with only modest changes in discrimination, and the analyses of detection timing, sex, and clinical presentation did not demonstrate clear differential effects. These findings should therefore be interpreted primarily as associations observed within a selected angiographic cohort rather than as evidence of causation or prospective predictive performance. Further prospective, multicenter studies with metabolic assessment before or at the time of PCI, standardized follow-up, and external validation are required to determine whether these indices have a role in routine ISR risk stratification.

## Data Availability

The original contributions presented in the study are included in the article/**Supplementary Material**. Further inquiries can be directed to the corresponding authors.

## ANNEX:

1. Cases selection.
2. Propensity score matching table.
3. Collinearity assessment results.
4. ISR occurrence supplementary analyses tables.
5. ISR severity supplementary analyses tables.
6. ISR angiographic detection supplementary analyses.
7. Sex based Subgroup analyses.
