# Supplementary material for: Association of metabolic indices with in-stent restenosis and its angiographic severity after drug-eluting stent implantation: a case-control study
Source: Front Endocrinol (Lausanne). 2026 Sep 21;17:1952213. doi: 10.3389/fendo.2026.1952213 (PMC13634957; doi:10.3389/fendo.2026.1952213)
Supplement: Supplementary file 1 [file SupplementaryFile1.docx]

**ANNEXES**

1.**Case selection flow and Analysis framework**

**Supplementary Figure**

**A**: CONSORT/STROBE style case-selection flowchart detailing the exact numbers from the initial angiogram query (N=15,600), down to the eligible unmatched cohort (N=1,650), and finally the 1:1 propensity-score matched cohort (N=774). **B:** Detailed analysis process including the different cohorts used for each analysis.

2. **Covariate Balance Before and After Propensity Score Matching**

| Covariate | SMD Before Matching | SMD After Matching | Post-Matching Balance |
| --- | --- | --- | --- |
| Age, years | 0.016 | 0.045 | Adequate |
| Sex | 0.041 | 0.017 | Adequate |
| Hypertension | 0.016 | 0.055 | Adequate |
| Diabetes mellitus | **0.738** | **0.006** | Adequate |
| Current smoking | 0.008 | 0.016 | Adequate |
| Statin use | **0.101** | 0.047 | Adequate |
| Gensini score | 0.014 | 0.048 | Adequate |
| Stent length, mm | **0.579** | **0.091** | Adequate |
| Vessel size, mm | **0.892** | **0.111** | Minor residual imbalance |
| Maximum absolute SMD | **0.892** | **0.111** | — |

3. **COLINEARITY ASSESSMENT**

GVIF Df GVIF^(1/(2*Df))

Diabetes	2.139088 1	1.462562

Current_Smoker	1.056134 1	1.027684

Stent_Length_mm	1.091883 1	1.044932

Vessel_Size_mm	1.467803 1	1.211529

Age_years	1.037719 1	1.018685

Hypertension	1.033273 1	1.016501

Bifurcation_Lesion	1.048079 1	1.023757

Location	1.087057 4	1.010489

Post_Dilatation	1.031476 1	1.015616

HDL_C_mg_dL	1.088082 1	1.043112

Fasting_Glucose_mg_dL	2.019832 1	1.421208

WBC	1.019366 1	1.009636

Gensini_Score	1.019206 1	1.009557

IVUS_OCT_Used	1.013894 1	1.006923

FIBRATES	1.024043 1	1.011950

No VIF > 4. Multicollinearity is not a major concern.

Below are the full multivariable logistic regression tables for the three defined outcomes: **ISR Occurrence**, **ISR Severity**, and **ISR Timing (Early vs. Late)**.

The tables display the Odds Ratios (OR), 95% Confidence Intervals (CI), and p-values for the standard baseline clinical model. Below the baseline clinical variables in each table, the independent effects of the three metabolic indices are provided. *(Note: The metabolic indices were evaluated individually by adding them one at a time to the baseline clinical model).*

3. **ISR occurrence supplementary analyses tables**

**(Matched Cohort, N=774)**

**Outcome:** In-Stent Restenosis (ISR) vs. No ISR.

| Covariate | Odds Ratio (OR) | 95% CI | P-value |
| --- | --- | --- | --- |
| Baseline Clinical Variables |  |  |  |
| Age (years) | 1.00 | 0.99 – 1.02 | 0.606 |
| Sex (Male) | 1.00 | 0.74 – 1.37 | 0.985 |
| Hypertension | 0.94 | 0.69 – 1.28 | 0.696 |
| Diabetes | 1.19 | 0.86 – 1.65 | 0.282 |
| Current Smoker | 1.01 | 0.75 – 1.36 | 0.948 |
| Statin Use | 1.14 | 0.50 – 2.59 | 0.760 |
| Stent Length (mm) | 1.02 | 1.00 – 1.03 | **0.045*** |
| Vessel Size (mm) | 0.69 | 0.48 – 0.98 | **0.041*** |
| Metabolic Indices (Added Individually) |  |  |  |
| + TyG Index | 2.16 | 1.59 – 2.95 | **<0.001*** |
| + AIP | 7.17 | 3.58 – 14.35 | **<0.001*** |
| + METS-IR | 1.02 | 1.00 – 1.04 | 0.124 |

**Tab Conditional logistic regression in the propensity-score-matched cohort**

| Metabolic Index | Matched N | Matched Pairs | Adjusted OR (95% CI) | P-value |
| --- | --- | --- | --- | --- |
| TyG | 774 | 387 | 2.07 (1.22–3.54) | 0.007 |
| AIP | 774 | 387 | 5.40 (1.76–16.56) | 0.003 |
| METS-IR | 774 | 387 | 1.02 (0.98–1.05) | 0.375 |

**Supplementary Table. Matched-Pair Bootstrap Validation of Incremental Discrimination**

| Model comparison | ΔAUC | Matched-pair bootstrap 95% CI | Paired DeLong P-value | Interpretation |
| --- | --- | --- | --- | --- |
| Baseline vs +TyG | +0.040 | 0.016 to 0.086 | 0.003 | Incremental discrimination supported |
| Baseline vs +AIP | +0.056 | 0.030 to 0.109 | <0.001 | Incremental discrimination supported |
| Baseline vs +METS-IR | 0.000 | −0.006 to 0.028 | 0.642 | No evidence of incremental discrimination |
| AIP vs TyG | +0.016 | −0.002 to 0.040 | 0.032 | DeLong test significant, but bootstrap CI includes zero |

_AUC, area under the receiver operating characteristic curve; ΔAUC, difference in AUC; TyG, triglyceride-glucose index; AIP, atherogenic index of plasma; METS-IR, metabolic score for insulin resistance; CI, confidence interval._

Differences in AUC were evaluated using 1,000 matched-pair bootstrap resamples. Bootstrap confidence intervals excluded zero for the addition of TyG and AIP to the baseline model, but not for METS-IR. Although the paired DeLong comparison between AIP and TyG yielded p=0.032, the corresponding bootstrap 95% CI included zero, indicating that evidence for superiority of AIP over TyG was not robust across resampling.

**4. ISR severity supplementary analyses tables**

**(Matched Cohort, N=387)**

**Outcome:** Very Severe ISR vs. Less Severe ISR *(restricted to patients who experienced an* **event).**

| Covariate | Odds Ratio (OR) | 95% CI | P-value |
| --- | --- | --- | --- |
| Baseline Clinical Variables |  |  |  |
| Age (years) | **0.99** | **0.97 – 1.01** | **0.285** |
| Sex (Male) | **1.10** | **0.71 – 1.72** | **0.663** |
| Hypertension | **1.05** | **0.68 – 1.63** | **0.815** |
| Diabetes | **0.95** | **0.60 – 1.51** | **0.823** |
| Current Smoker | **0.96** | **0.63 – 1.46** | **0.845** |
| Statin Use | **1.06** | **0.31 – 3.61** | **0.923** |
| Stent Length (mm) | **1.02** | **0.99 – 1.04** | **0.171** |
| Vessel Size (mm) | **0.83** | **0.53 – 1.29** | **0.403** |
| Metabolic Indices (Added Individually) |  |  |  |
| + TyG Index | 1.55 | 1.00 – 2.40 | 0.051 (Borderline) |
| + AIP | 3.06 | 1.18 – 7.94 | 0.021* |
| + METS-IR | 1.00 | 0.97 – 1.03 | 0.882 |

**Multivariable multinomial logistic regression for ISR severity**

Reference category: **No ISR**

| Comparison | Metabolic Index | Adjusted OR (95% CI) | P-value |
| --- | --- | --- | --- |
| Less Severe ISR vs No ISR | **TyG** | **1.06 (0.53–2.15)** | **0.865** |
|  | **AIP** | **3.44 (0.69–17.01)** | **0.130** |
|  | **METS-IR** | **1.00 (0.98–1.03)** | **0.785** |
| Very Severe ISR vs No ISR | **TyG** | **1.02 (0.49–2.13)** | **0.952** |
|  | **AIP** | **14.93 (2.75–81.01)** | **0.002** |
|  | **METS-IR** | **0.99 (0.96–1.02)** | **0.654** |

All three metabolic indices were included simultaneously, and the model was additionally adjusted for age, sex, hypertension, diabetes mellitus, smoking status, statin use, stent length, and vessel size.

**Direct comparison of Very Severe vs Less Severe ISR**

| Metabolic Index | Adjusted OR (95% CI) | P-value |
| --- | --- | --- |
| TyG | 0.96 (0.43–2.18) | 0.926 |
| AIP | 4.34 (0.66–28.45) | 0.126 |
| METS-IR | 0.99 (0.96–1.02) | 0.525 |

This second contrast is particularly important. Although AIP was strongly associated with the Very Severe ISR category relative to controls, its OR for Very Severe vs Less Severe ISR was 4.34 but had a very wide CI and was not statistically significant. Therefore, the data do not establish that AIP independently determines greater angiographic severity once ISR is already present.

Multinomial analysis of ISR severity. In a mutually adjusted multinomial logistic regression model including TyG, AIP, and METS-IR simultaneously, with No ISR as the reference category, none of the metabolic indices was independently associated with Less Severe ISR. For Very Severe ISR, AIP remained independently associated with the outcome (adjusted OR 14.93, 95% CI 2.75–81.01; p=0.002), whereas TyG (adjusted OR 1.02, 95% CI 0.49–2.13; p=0.952) and METS-IR (adjusted OR 0.99, 95% CI 0.96–1.02; p=0.654) were not. However, in the direct comparison of Very Severe versus Less Severe ISR, none of the three metabolic indices was statistically significant: TyG OR 0.96 (95% CI 0.43–2.18; p=0.926), AIP OR 4.34 (95% CI 0.66–28.45; p=0.126), and METS-IR OR 0.99 (95% CI 0.96–1.02; p=0.525).

These findings suggest that AIP may be associated with membership in the very-severe ISR category when compared with patients without ISR. However, the absence of a statistically significant association in the direct Very Severe versus Less Severe ISR comparison indicates that AIP should not be interpreted as an independent marker of ISR severity progression. The wide confidence interval also indicates substantial uncertainty. TyG and METS-IR did not show independent associations after simultaneous adjustment for the three correlated metabolic indices.

One additional point: because TyG, AIP, and METS-IR are highly correlated, the simultaneous model answers a different question from the separate models. It asks whether each index contributes information independent of the other two. The loss of the TyG association is therefore not contradictory; it suggests its association overlaps considerably with information captured by AIP and METS-IR.

**5. ISR angiographic detection supplementary table**

**(ISR Cohort, N=820)**

Outcome: Early ISR (≤12 months) vs. Late ISR (>12 months).

| Covariate | Odds Ratio (OR) | 95% CI | P-value |
| --- | --- | --- | --- |
| Baseline Clinical Variables |  |  |  |
| Age (years) | **0.99** | **0.97 – 1.01** | **0.295** |
| Sex (Male) | **0.95** | **0.68 – 1.33** | **0.765** |
| Hypertension | **1.00** | **0.73 – 1.38** | **0.988** |
| Diabetes | **0.85** | **0.61 – 1.20** | **0.365** |
| Current Smoker | **0.96** | **0.70 – 1.30** | **0.783** |
| Statin Use | **1.94** | **0.54 – 6.93** | **0.307** |
| Stent Length (mm) | **1.00** | **0.98 – 1.01** | **0.738** |
| Vessel Size (mm) | **1.18** | **0.85 – 1.65** | **0.324** |
| Metabolic Indices (Added Individually) |  |  |  |
| + TyG Index | **1.23** | **0.90 – 1.68** | **0.188** |
| + AIP | **1.09** | **0.56 – 2.10** | **0.806** |
| + METS-IR | **0.99** | **0.97 – 1.02** | **0.635** |

**6. Formal sex-interaction tests**

| Outcome | Metabolic Index | P for Interaction | BH-Adjusted P |
| --- | --- | --- | --- |
| ISR Severity | TyG | 0.630 | 0.630 |
| ISR Severity | AIP | 0.503 | 0.630 |
| Timing of ISR Detection | TyG | 0.385 | 0.385 |
| Timing of ISR Detection | AIP | 0.228 | 0.385 |

There was no statistically supported sex heterogeneity for either ISR severity or timing
